# Nuclear Eg5 (kinesin spindle protein) expression predicts docetaxel response and prostate cancer aggressiveness

**DOI:** 10.18632/oncotarget.1985

**Published:** 2014-05-16

**Authors:** Michel D. Wissing, Ellen S. De Morrée, Vincent O. Dezentjé, Jeroen T. Buijs, Ronald R. De Krijger, Vincent T.H.B.M. Smit, Wytske M. Van Weerden, Hans Gelderblom, Gabri van der Pluijm

**Affiliations:** ^1^ Department of Clinical Oncology, Leiden University Medical Center, Albinusdreef 2, 2333 ZA Leiden, the Netherlands; ^2^ Department of Urology, Leiden University Medical Center, Albinusdreef 2, 2333 ZA Leiden, the Netherlands; ^3^ Department of Urology, Erasmus MC-Cancer Institute, ‘s-Gravendijkwal 230, 3015 CE Rotterdam, the Netherlands; ^4^ Department of Pathology, Reinier de Graaf Gasthuis, Reinier de Graafweg 3, 2625 AD Delft, the Netherlands; ^5^ Department of Pathology, Leiden University Medical Center, Albinusdreef 2, 2333 ZA Leiden, the Netherlands

**Keywords:** biomarker, docetaxel, Eg5, kinesin spindle protein, prostate cancer

## Abstract

Novel biomarkers predicting prostate cancer (PCa) aggressiveness and docetaxel therapy response of PCa patients are needed. In this study the correlation between nuclear Eg5-expression, PCa docetaxel response and PCa aggressiveness was assessed. Immunohistochemical staining for nuclear Eg5 was performed on 117 archival specimens from 110 PCa patients treated with docetaxel between 2004 and 2012. Samples were histologically categorized as positive/negative.

Median follow-up time from diagnosis was 11.6 years. Nuclear Eg5-expression was significantly related to docetaxel response (p=0.036) in tissues acquired within three years before docetaxel initiation. Nuclear Eg5-expression was not related to Gleason-score (p=0.994). Survival of patients after docetaxel initiation did not differ based on nuclear Eg5-expression (p=0.540). Analyzing samples taken before hormonal therapy, overall survival and time to docetaxel use were significantly decreased in patients with nuclear Eg5-expressing tumors (p<0.01). Eg5-positive nuclei were found more frequently in T4-staged tumors (p=0.04), Gleason 8-10 tumors (p=0.08), and in metastasized tumors (p<0.01). Multivariate analyses indicated that nuclear Eg5-expression may be an independent parameter for tumor aggressiveness. Limitations of a retrospective analysis apply.

In conclusion, nuclear Eg5-expression may be a predictive biomarker for docetaxel response in metastatic castrate-resistant PCa patients and a prognostic biomarker for hormone-naive PCa patients. Prospective validation studies are needed.

## INTRODUCTION

Metastatic castrate-resistant prostate cancer (mCRPC) is the second deadliest cancer in men in the Western world [1]. Primary first-line therapy for most mCRPC patients consists of the taxane docetaxel with prednisone [2;3], although various other mCRPC therapies have recently been introduced [4-8]. About 48% of patients initially respond to docetaxel therapy [2]; eventually all patients progress during or after docetaxel, usually within few months after their last cycle. As docetaxel inhibits depolarization of microtubules regardless of cell type [9], toxicities may be severe, such as polyneuropathy and bone marrow suppression [2]. To prevent or restrict unnecessary docetaxel use, and to determine the optimal treatment sequence for individual mCRPC patients [10], biomarkers predicting docetaxel response need to be identified and implemented in clinical practice [11].

We hypothesized that nuclear Eg5 (Kindle Spindle Protein/KSP/KIF11/kinesin-5) may be such a marker. Eg5 separates spindle poles of a mitotic cell by crosslinking two antiparallel microtubules and moving to the plus-ends of both microtubules [12]. Due to its essential function in mitosis, multiple Eg5-inhibitors have been developed for anti-cancer therapy, such as ispinesib [13]. Two studies with ispinesib focused particularly on mCRPC patients, with ambiguous results. In a phase I study, six out of fourteen mCRPC patients had stable disease (SD) for ≥18 weeks and one patient had a prostate-specific antigen (PSA)-decrease of >50% when ispinesib was combined with docetaxel in mCRPC patients [14]. In a phase II study in which ispinesib was administered as monotherapy, no responses were reported [15]. Twenty out of 21 patients had been treated with docetaxel prior to ispinesib. Immunohistochemistry analysis on archival tumor tissue from sixteen patients indicated that only one tumor stained positive for Eg5. It was concluded that ispinesib is not effective in primary prostate cancer (PCa) due to their low mitotic index, resulting in low Eg5 expression. However, considering their similar mechanism of action, an alternative explanation could be that cross-resistance occurs between docetaxel and Eg5-inhibitors.

Recent studies indicate that Eg5 may also play a role in intracellular transport in the cytoplasm, suggesting that Eg5-inhibitors may target Eg5 expressing non-mitotic cells too [16;17]. Xing et al. analyzed archival specimens from 80 patients with clinically localized PCa; half stained positive for Eg5, while benign prostate cells did not express Eg5 [18]. Considering the low mitotic index of PCa cells regardless of disease stage [19], these data suggest that Eg5 may indeed be expressed in non-mitotic PCa cells too [20].

Combining aforementioned findings [14;15;18], initial Eg5 expression of PCa may have been decreased once tumors have become docetaxel resistant. This led to our hypothesis that Eg5 may be a predictive marker for docetaxel response. Based on recent findings that patients with high Gleason-scores respond better to taxane-based therapy [21], we further hypothesize that Eg5 may be a prognostic marker for tumor aggressiveness and clinical outcome.

## RESULTS

### Patient and tissue characteristics

In total, 117 samples were collected from 110 mCRPC patients. These patients had been diagnosed with PCa between 1994 and 2011 and treated with docetaxel between July 15th, 2004 and December 24th, 2012. Median time to follow-up from date of PCa diagnosis was 11.6 years (interquartile range 8.7-14.2 years). Clinicopathological parameters are listed in Table 1. Median age of patients when diagnosed with PCa was 64 years. Median Gleason-score of tumors was 8. About two-thirds of patients had ≥2 measured metastatic localizations when docetaxel was initiated. Of note, tumor imaging methods such as CT-scans were not performed in all patients, underestimating the number of metastatic lesions. All patients had been medically and/or surgically castrated. In general, patients had been heavily pretreated: patients had received up to five therapies before docetaxel therapy.

**Table 1:** Characteristics of mCRPC patients, their disease and treatment (n=110), and of the obtained tissue (n=117)

Patient Age				
	At time of prostate cancer diagnosis [median (range)]		64	(43-84)
	At time of obtaining tissue [median (range)]		65	(43-86)
	At time of start docetaxel [median (range)]		69	(46-87)
				
Disease characteristics (diagnostic imaging)				
	Gleason score [median (range)]			
		All patients	8	(4-10)
		Hormone-naive patients	8	(4-10)
	Number of metastatic lesions [number of patients (%)]			
		1	37	(33.6%)
		2	49	(44.5%)
		≥3	24	(21.8%)
	Localization of metastases [number of patients (%)]			
		Lymph node	71	(64.5%)
		Bone	106	(96.4%)
		Liver	10	(9.1%)
		Lung/pleura	16	(14.5%)
		brain	1	(0.9%)
				
Treatment characteristics				
	Pretreatment [number of patients (%)]			
		Androgen-deprivation therapy	109	(99.1%)
		Radical prostatectomy	15	(13.6%)
		TUR-P	25	(22.7%)
		Surgical castration	4	(3.6%)
		Lymph node dissection	34	(30.9%)
		Radiotherapy prostate	34	(30.9%)
		Radiotherapy metastases	40	(36.4%)
		Other	4	(3.6%)
	Docetaxel treatment			
		# courses [median (range)]	1	(1-3)
		# cycles [median (range)]	6	(1-20)
		Best response [number of patients (%)]:		
		progressive disease	22	(20.0%)
		stable disease	38	(34.5%)
		partial response	49	(44.5%)
		Docetaxel rechallenge [number of patients (%)]	7	(6.4%)
	Posttreatment [number of patients (%)]		91	(82.7%)
		Cabazitaxel	16	(14.5%)
		Abiraterone	30	(27.3%)
		Enzalutamide	6	(5.5%)
		Radiotherapy	47	(42.7%)
		Strontium-89	24	(21.8%)
		Samarium-153	4	(3.6%)
		Mitoxantrone	15	(13.6%)
		Other	8	(7.3%)
				
Obtained pathological material				
	Type of material [number of samples (%)]			
		Biopsy	82	(70.0%)
		TUR-P	24	(20.5%)
		Radical prostatectomy	11	(9.4%)
	Disease stage [number of samples (%)]			
		hormone-naive	87	(74.4%)
		pre-docetaxel	112	(95.7%)
		within three years of start docetaxel	61	(52.1%)
		mCRPC post-docetaxel	5	(4.3%)
				
Survival				
	OS in years [median (IQR)]		4.8	(2.6-9.3)
	Lost-to-follow-up [number of patients (%)]		18	(16.4%)

IQR, interquartile range; OS, overall survival; TUR-P, transurethral resection of the prostate

**Figure 1:**
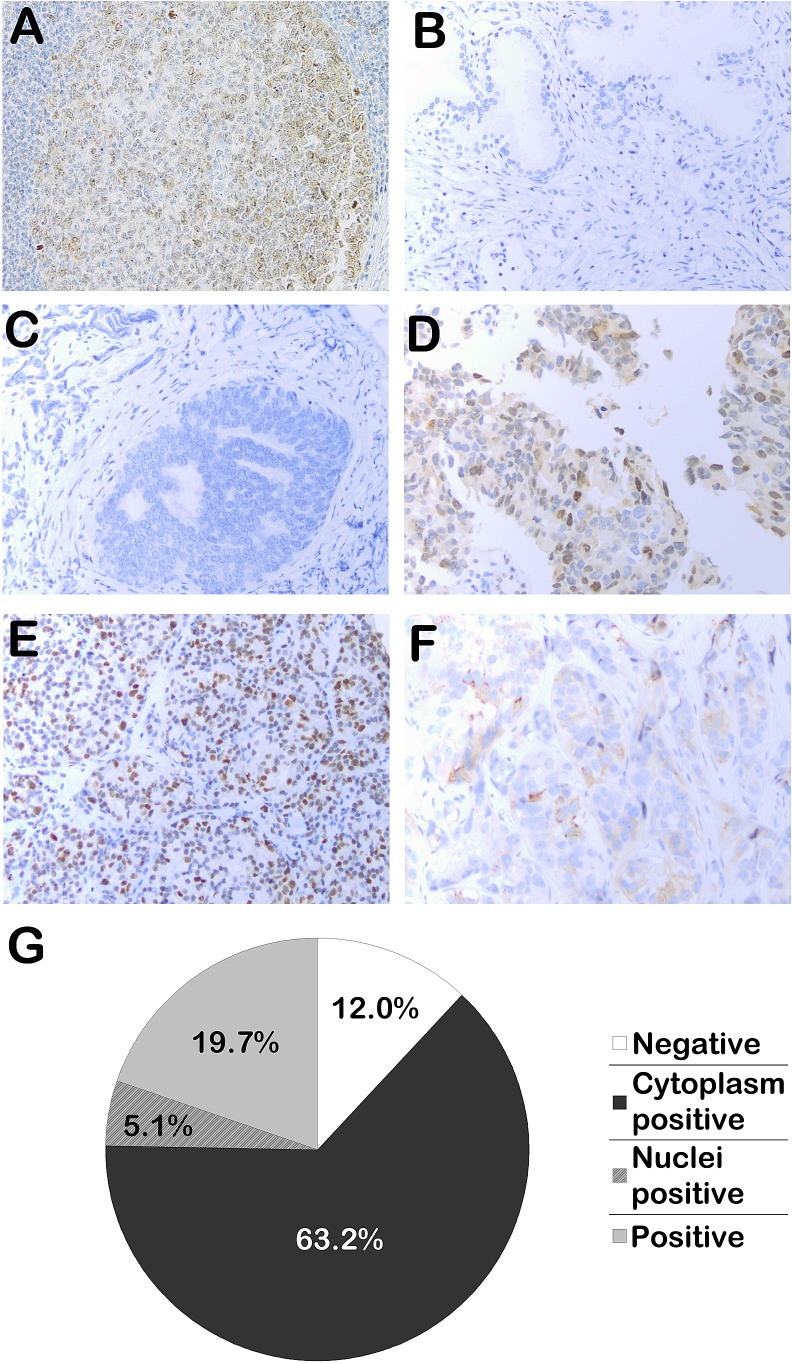
Immunohistochemical analysis of Eg5 expression in human clinical samples A. Positive control: a lymphatic tissue in a tonsil. B. Negative control: healthy prostate tissue. C. Prostate cancer (PCa) sample staining negative for Eg5. D. PCa sample with Eg5 expression in both the nuclei and cytoplasm. E. PCa sample with nuclear Eg5 expression. F. PCa sample with cytoplasmic Eg5 expression. G. Percentages indicate the frequencies samples with this subcellular staining pattern were found in our sample set (n=117).

For immunohistochemistry, tonsil and healthy prostate tissue served as positive and negative controls, respectively (Fig. 1A-B). Obtained PCa tissue consisted primarily of biopsies (70.0%) (Table 1, supplementary Fig. 1). In the tumor samples, a clear distinction was observed between samples with nuclear Eg5 staining (5.1%), cytoplasmic Eg5 staining (19.7%), and samples staining positive for Eg5 in both compartments (63.2%), irrespective of the samples' age (Fig. 1C-F). Samples were scored for nuclear or cytoplasm staining separately. Interobserver agreement of scoring was 98.1%.

### Immunohistochemical Eg5 expression and docetaxel response

**Figure 2:**
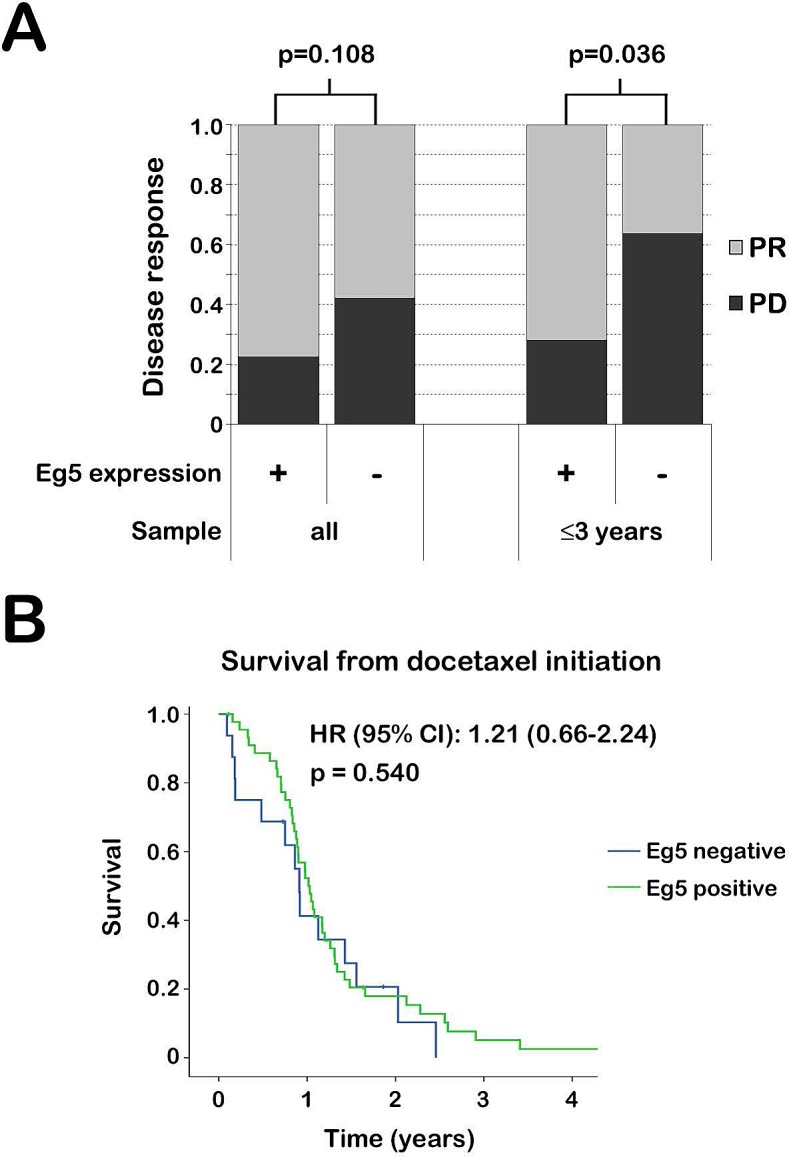
Nuclear Eg5 expression and docetaxel response in mCRPC patients A. Best disease response to docetaxel therapy in mCRPC patients, grouped by nuclear Eg5 expression of their tumor. Patients with stable disease (SD) were excluded from this analysis. The most recent PCa tissue before docetaxel therapy was analyzed from all patients (left) or only from patients who had tissue available obtained from the patient within three years before docetaxel therapy (right). In general, patients with nuclear Eg5 expression had a higher percentage of partial responses (PR). PD, progressive disease. B. Overall survival (OS) after docetaxel initiation. Patients were excluded when they only had PCa tissue available acquired more than three years before docetaxel therapy. Selected mCRPC patients were grouped based on nuclear Eg5 expression of their tumor. Median OS did not differ between patient groups, although initially there is more patient death in the group with Eg5-negative tumors.

Eg5 expression varied in tumors from some patients who had multiple biopsies taken before docetaxel therapy. This variability always reflected a disappearance of Eg5 expression over time. It is unknown whether these changes occurred as the tumor evolved spontaneously or due to other therapies, such as androgen-deprivation therapy. Therefore, correlation between Eg5 expression and docetaxel response was evaluated for all patients (n=110) as well as for patients with samples taken within three years before docetaxel start (n=61). A clear trend was observed between nuclear Eg5 expression and a better response to docetaxel therapy (Fig. 2A, Supplementary Fig. 2). This correlation was significant in patients from whom tissue was taken within three years before docetaxel initiation: 71.9% of these patients with nuclear Eg5 expression had a PR versus 36.4% of patients without nuclear Eg5 expression (p=0.036). Conversely, cytoplasmic or any Eg5 expression did not predict docetaxel response (Supplementary Fig. 3).

As a previous report identified Gleason-scores as a predictive marker for docetaxel response, it was tested whether a correlation existed between Gleason-score and docetaxel response in our set of patient samples (Supplementary Table 1). Gleason-score was not related to docetaxel response, neither in all patients (p=0.343) nor in patients with tissue available in the three years before docetaxel initiation (p=0.884). Furthermore, Gleason-score and nuclear Eg5 expression were not related in this latter subpopulation (p=0.994), suggesting that nuclear Eg5 expression was an independent marker of docetaxel response.

We further explored the correlation between docetaxel response and Eg5 expression by investigating patients who had a PCa sample taken before and after docetaxel treatment. Only five patients matched these criteria. While cytoplasmic Eg5 expression did not alter in these patients, three out of four tumors with positive Eg5 nuclei before docetaxel therapy did not have nuclear Eg5 expression after docetaxel treatment (Supplementary Figure 4). These three patients had progressive disease upon discontinuation of docetaxel. On the other hand, the patient whose tumor expressed nuclear Eg5 pre- and post-docetaxel discontinued docetaxel therapy due to unacceptable toxicities. Despite the small patient number, these results suggested that loss of nuclear Eg5 expression may be related to docetaxel resistance.

Intriguingly, although patients with nuclear Eg5 expression had a better response to docetaxel (Fig. 2A), no difference in OS, calculated from the start of docetaxel therapy to death, was evident between tumors based on nuclear Eg5 expression (p=0.540) (Fig. 2B).

### Immunohistochemical nuclear Eg5 expression and tumor aggressiveness

**Figure 3:**
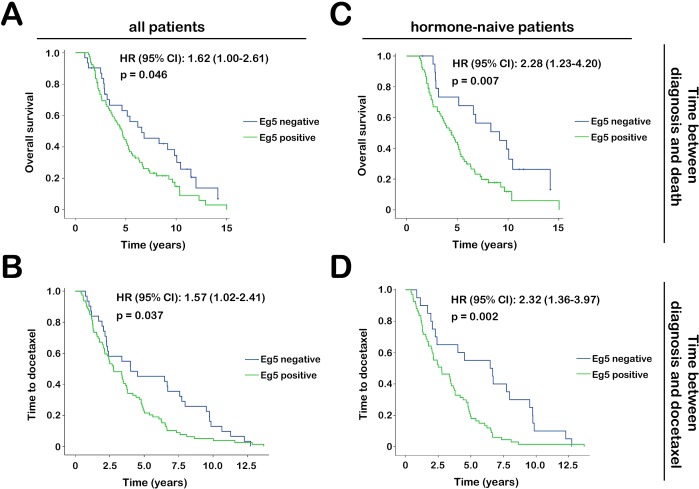
Tumor aggressiveness in mCRPC patients based on Eg5 expression Patients were selected of whom PCa tissue acquired within three years (left) or three months (right) of diagnosis was available. Patients were divided in groups based on nuclear Eg5 expression. Median OS (top) and time to symptomatic mCRPC (bottom) were compared between patients groups. Patients with tumors that expressed Eg5 upon diagnosis, had a worse clinical outcome.

**Figure 4:**
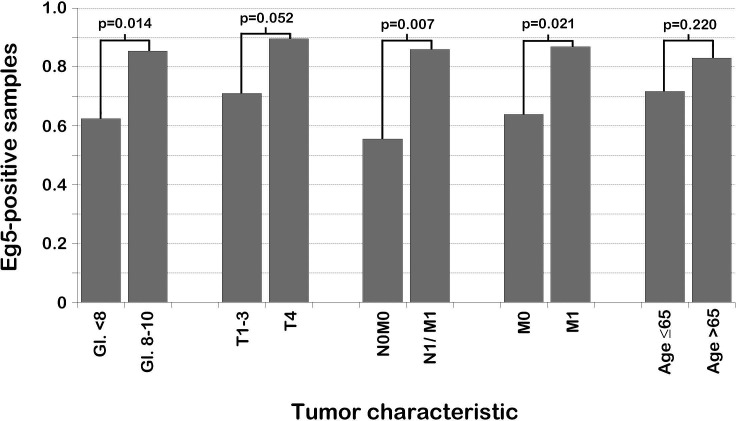
Correlation between PCa characteristics and nuclear Eg5 expression in PCa patients Patients were selected who had PCa tissue available within three years of PCa diagnosis. Tumors were divided in groups based on Gleason-score and TNM-classification upon diagnosis; the percentage of tumors with Eg5 expressing nuclei was compared. In general, PCa with nuclear Eg5 expression was more aggressive. Gl, Gleason; T, tumor stage according to TNM-classification; N, lymph node metastases (0, no metastases; 1, metastases); M, distant metastases (0, no metastases; 1, metastases), N1/M1, any metastases (lymph node and/or distant metastases).

We evaluated whether tumors with nuclear Eg5 expression behaved more aggressively. Analyzing samples from all 110 patients, patients with tumors with nuclear Eg5 expression had a significantly decreased OS (median 6.6 versus 4.7 years, p=0.046) (Fig. 3A). Time from diagnosis to symptomatic mCRPC was also decreased (median 4.0 versus 2.8 years, p=0.037) (Fig. 3B). When selecting samples from hormone-naive patients (n=87), differences in OS and time to symptomatic mCRPC were even more pronounced (p=0.010 and p=0.006, respectively) (Fig. 3C-D). In this subset of patients, nuclear Eg5 expression was related to Gleason-score (p=0.014) and TNM classification (tumor stage, p=0.052; any metastases, p=0.007; distant metastases, p=0.021); no correlation existed between nuclear Eg5 expression and age (Fig. 4).

Multivariate analyses were performed to test whether the correlation between nuclear Eg5 expression and tumor aggressiveness (OS and time to symptomatic mCRPC) remained evident when correcting for potential confounding variables, such as Gleason-score (Table 2). When including all patients, addition of most covariates resulted in no statistically significant correlation between nuclear Eg5 expression and OS or time to symptomatic mCRPC. This included correction for age, while this variable was neither related to nuclear Eg5 expression nor to prognosis, suggesting the study was underpowered for such analyses. However, a trend towards positive nuclear Eg5 expression and aggressive tumors was evident. When assessing hormone-naive patients, a clearly positive trend existed between nuclear Eg5 expression and tumor aggressiveness regardless of the covariate added (hazard ratio >1.75), suggesting a potential independent prognostic value for nuclear Eg5 expression. The correlation between nuclear Eg5 expression and time to symptomatic mCRPC was significant in all subgroup analyses, except when metastases (N1 and/or M1) were added as a covariate (p=0.063).

**Table 2:** Multivariate analysis using the Cox-regression model exploring potential confounders for the correlation between nuclear Eg5 expression and tumor aggressiveness

	All patients				Hormone naive patients			
Covariate	OS [HR (95% CI)]			TTH [HR (95% CI)]			OS [HR (95% CI)]			TTH [HR (95% CI)]	
Age	1.56	(0.95-2.54)		1.56	(1.00-2.42)		2.13	(1.13-4.02)		2.29	(1.31-3.98)
Gleason	1.48	(0.91-2.40)		1.45	(0.94-2.23)		1.76	(0.92-3.36)		1.78	(1.00-3.18)
Gleason <7 and ≥7	1.58	(0.95-2.63)		1.50	(0.95-2.38)		2.61	(1.35-5.07)		2.67	(1.50-4.75)
Gleason <8 and ≥8	1.48	(0.91-2.40)		1.46	(0.95-2.26)		1.84	(0.79-3.49)		1.93	(1.09-3.41)
T stage	1.75	(1.03-2.99)		1.59	(0.99-2.53)		2.56	(1.32-4.97)		2.64	(1.47-4.75)
Any metastases	1.52	(0.81-2.85)		1.28	(0.74-2.22)		2.25	(0.99-5.13)		1.97	(0.96-4.02)
Distant metastases	1.40	(0.79-2.49)		1.25	(0.75-2.08)		1.84	(0.87-3.87)		2.33	(1.14-4.79)
Number of metastases	1.64	(1.01-2.65)		1.59	(1.04-2.44)		2.61	(1.35-5.07)		2.67	(1.50-4.75)

CI, confidence interval; HR, hazard ratio; OS, overall survival; T stage, T stage according to TNM classification; TTD, time to docetaxel therapy.

## DISCUSSION

Research has been ongoing identifying prognostic biomarkers and biomarkers predictive for therapy response in PCa with improved accuracy compared to established biomarkers such as serum PSA levels and Gleason-score, with some success [11]. Urokinase plasminogen activator and its inhibitor PAI-1, and Ki-67 have been identified as potential prognostic biomarkers of PCa [22;23]. Cytoplasmic localization of the androgen-receptor and increased blood serum levels of Macrophage Inhibitory Cytokine 1 (MIC-1) have been identified as a potential marker for PCa docetaxel response [24;25]. PCa tumors expressing class III beta-tubulin were relatively insensitive to PCa therapy: class III beta-tubulin expression resulted in faster recurrence after radical prostatectomies, a decreased docetaxel response and decreased survival [26]. Unfortunately, none of these markers are available yet for use in clinical practice [11]. Additional studies, such as the one we present here, are needed to identify a biomarker that is related to docetaxel response in PCa patients.

In the current study, we found that nuclear Eg5 expression in PCa was associated with improved antitumor efficacy of docetaxel, independently of patient's Gleason-score. Furthermore, we identified nuclear Eg5 as a prognostic marker in hormone-naive PCa patients: patients whose tumor expressed nuclear Eg5 had a decreased median OS and progressed more rapidly to mCRPC. Similar findings were reported in non-small lung cancer patients: patients with Eg5 expressing tumors had a better response to chemotherapy, but a lower OS [27]. Similarly, Eg5 expression was related to worse clinical outcome in renal cell carcinoma patients [28].

Once docetaxel was initiated, survival of mCRPC patients was similar irrespective of nuclear Eg5 expression. This may indicate that nuclear Eg5 expressing tumors initially respond well to docetaxel, resulting in decreased patient mortality. However, once these Eg5 expressing tumors progress, these tumors behave more aggressively, increasing patient death. This trend could indeed be derived from the survival curve (Fig. 2B) and might explain why survival of patients with nuclear Eg5 expression is not increased after docetaxel treatment despite responding better to docetaxel therapy. Alternatively, other factors may have resulted in the similar survival curve, such as unequal patient and treatment characteristics between groups other than Eg5 expression.

Nuclear Eg5 expression could provide a useful tool for clinical practice. Interobserver agreement between researchers was very high (98.1%), as no subjective degrees of positive staining (mild/moderate/strong) were used. Positive/negative scoring requires little interpretation from the pathologist. Determination of Eg5 expression at the time of diagnosis would be non-invasive, as tissue material is already acquired. Additional tissue sampling once the mCRPC stage has been reached could aid the physician in deciding when to initiate docetaxel therapy. Patients whose tumor expresses nuclear Eg5 may benefit by early docetaxel treatment; patients with Eg5-negative tumors may be recommended to initiate other therapies first, as docetaxel response is more limited.

In the current study, a retrospective design was chosen, resulting in several limitations. FFPE PCa samples were collected from pathology archives; these samples were taken for diagnostic purposes (biopsies) and consisted of residual materials from surgical procedures such as TUR-P or radical prostatectomies. Therefore, the sample set we created was heterogeneous in origin. However, on the contrary to other tumors such as breast cancer, only limited tissue material is available from PCa patients during their disease, as many patients have a prostatectomy early in their disease and primarily suffer from bone metastases, which are not easily accessible. Furthermore, additional tissue sampling is often not needed, as it currently would not influence further therapy decisions. Therefore, although our initial patient number was relatively large, only limited tissue material was available from patients shortly before docetaxel initiation. Hence all samples taken within three years of docetaxel initiation were collected for analysis. This led to a heterogeneous cohort of samples (both biopsies and residual surgical material), representing various stages of PCa disease. Furthermore, patients may have received various treatments between tissue sampling and docetaxel initiation. E.g., antiandrogen treatment significantly changed gene expression profiles of prostate cancer [29]. It is unknown whether such treatment specifically affects nuclear Eg5 expression. To overcome these challenges, a prospective study will be needed in which tissue will be collected shortly before docetaxel initiation to confirm the correlation between nuclear Eg5 expression and docetaxel response. However, such a study will need to overcome ethical and practical challenges as well.

In addition, our patient population was underpowered for multivariate analyses in hormone-naive patients. Further prospective studies are warranted to validate whether nuclear Eg5 expression may serve as an independent prognostic biomarker.

Previous studies found that PCa patients with aggressive tumors respond well to docetaxel, but also respond better to cabazitaxel, suggesting that aggressive tumors respond well to taxanes in general [30;31]. Therefore, additional studies are needed to assess whether Eg5 predicts response to cabazitaxel too. Finally, our study results suggest that loss of Eg5 expression may be related to docetaxel resistance. Although ispinesib had limited antitumor efficacy after docetaxel, our study and previous phase I findings suggest that ispinesib may be effective when administered before or concomitantly with docetaxel, when up to 70% of tumors express nuclear Eg5 [14]. However, combination therapy with docetaxel would need direct comparison to docetaxel monotherapy. Eg5-inhibitors may provide further clinical benefit when selecting mCRPC patients based on nuclear Eg5 expression (personalized medicine).

In conclusion, nuclear Eg5 expressing PCa is aggressive, but responds well to docetaxel. Loss of nuclear Eg5 expression may be associated with docetaxel resistance. Determining nuclear Eg5 expression in PCa samples may aid to improve timing to initiate docetaxel therapy in individual PCa patients. Additional prospective studies are needed to confirm the predictive and prognostic value of nuclear Eg5.

## METHODS

### Collection of patient material and data

Formalin fixed and paraffin embedded (FFPE) human PCa samples (biopsies, transurethral resections of prostate (TUR-P) or radical prostatectomies), stored at room temperature, were collected from pathology archives of Leiden University Medical Center, Reinier de Graaf Gasthuis and Erasmus Medical Center Rotterdam. mCRPC patients who had pathological material available taken before docetaxel therapy were included. The study was carried out in accordance with the Dutch code of conduct for the secondary use of human tissues; informed consent was therefore not required when enough material remained to serve the patient's and family's needs [32]. Additional patient information was collected anonymously in a database. Approval was obtained from the Medical Ethics Board (METC) of Leiden University Medical Center (P12.219).

### Immunohistochemistry

Samples (3μm sections) were stained for Eg5 using a polyclonal Anti-Eg5 antibody (1:1500, HPA006916, Sigma-Aldrich) on an automated immunohistochemistry stainer (Ventana Benchmark Ultra) (Fig. 1). This stainer utilized the ultraView Universal DAB Detection Kit (760-500, Ventana) for visualization of antibodies. The kit consisted of various enzyme labeled secondary antibodies that bind to primary antibodies; the complex was visualized with hydrogen peroxidase substrate and a 3.30-diaminobenzidine tetrahydrochloride (DAB) chromogen. For antigen retrieval, ULTRA CC1, an EDTA-Tris pH 8.4 solution, was used (950-224, Ventana). Representative images were taken at 20×10 under an Olympus BX41 microscope (Olympus Optical Co., Ltd.) from each slide using a colorview IIIu camera (Olympus), and analyzed with Cell^B imaging software (version 2.4108-181207). If an image was representative for the whole slide, only one picture was taken; otherwise, three representative views were imaged per slide.

### Data analysis

Images were examined and scored blindly and independently by two researchers (MDW, ESdM). A clear contrast between nuclear and cytoplasmic Eg5 staining was evident (Fig. 1). Recent studies have indicated that intracellular functions of Eg5 may differ based on its subcellular localization [17]; not all functions may be related to docetaxel response. Therefore, samples were scored for positive or negative staining of nuclei, cytoplasm or any cellular compartment (nucleus and/or cytoplasm).

Samples were considered positive when in one high-power field of view (20×10) at least four cancer cells were positive, regardless of intensity. This cut-off value ensured that random mitotic cells, infrequently found in the negative control too, were excluded. For analysis, average scores from both observers were calculated. If >50% of all scores per sample were positive for Eg5, the sample was considered Eg5-positive; otherwise it was considered Eg5-negative.

### Clinical endpoints

Clinical endpoints used in this study include survival from docetaxel initiation, overall survival (OS), time to symptomatic mCRPC and best therapy response.

Time to symptomatic mCRPC was defined as time between PCa diagnosis and docetaxel initiation. OS was calculated as time between diagnosis and patient death. If patients had not died or were lost to follow-up, survival was censored at the day the patient was last known to be alive before July 20th, 2013. Tumor aggressiveness was based on OS, time to symptomatic mCRPC, Gleason-score, and TNM-classification. Determination of best disease response (progressive disease, partial response) followed PCa working group guidelines as described previously, and could indicate PSA response and/or response as viewed on imaging such as computer tomography [30;33].

### Statistical analyses

Microsoft Excel 2003 was used for basic statistical analyses; student's t-tests were conducted for comparisons. SPSS (version 20) was used for the Kaplan-Meier analyses of survival and time to symptomatic mCRPC; log-rank tests were used to compare these parameters between groups. Multivariate analyses were performed using the Cox-regression model. P-values ≤0.050 were considered statistically significant.

## SUPPLEMENTARY DATA FIGURES AND TABLE


