# Supplementary material for: Nuclear Eg5 (kinesin spindle protein) expression predicts docetaxel response and prostate cancer aggressiveness
Source: Oncotarget. 2014 May 16;5(17):7357–67. doi: 10.18632/oncotarget.1985 (PMC4202128; doi:10.18632/oncotarget.1985)
Supplement: Supplementary file 1 [file oncotarget-05-7357-s001.doc]

**Supplementary data – Online only**

**
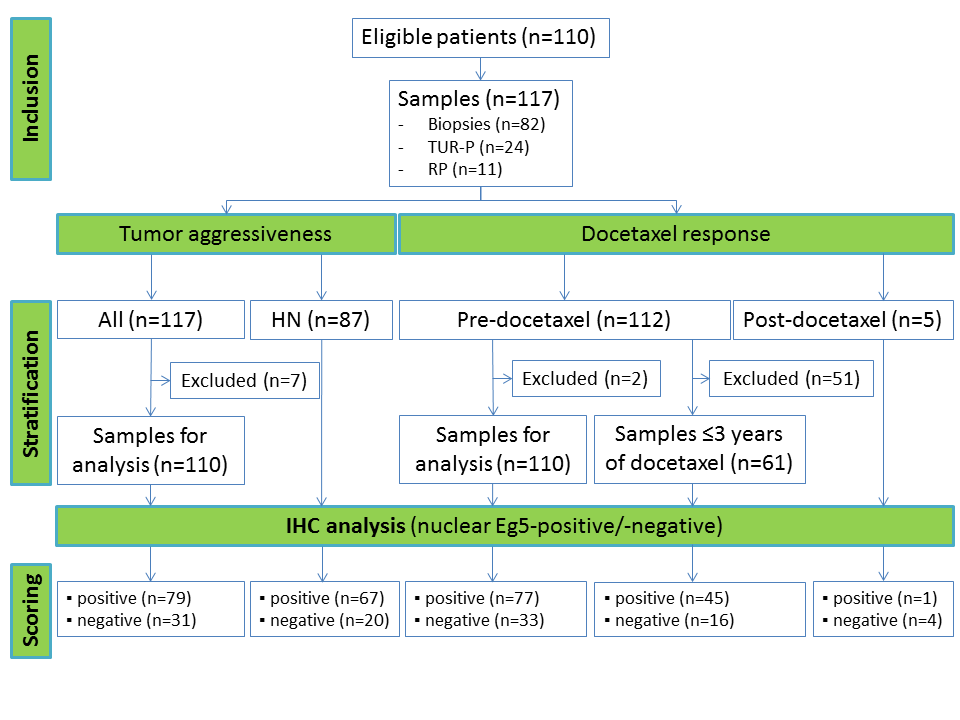
**

**Supplementary figure 1.** Diagram depicting sample size for all performed IHC analyses. Per patient, maximal one sample was included in each analysis: for tumor aggressiveness the patients’ sample that had the shortest duration between diagnosis and acquisition of tumor material (resulting in 7 exclusions), for docetaxel response the sample that was taken shortest before docetaxel was started (resulting in 2 exclusions). HN, hormone-naive; IHC, immunohistochemistry; RP, radical prostatectomy; TUR-P, transurethral resection of prostate.

**
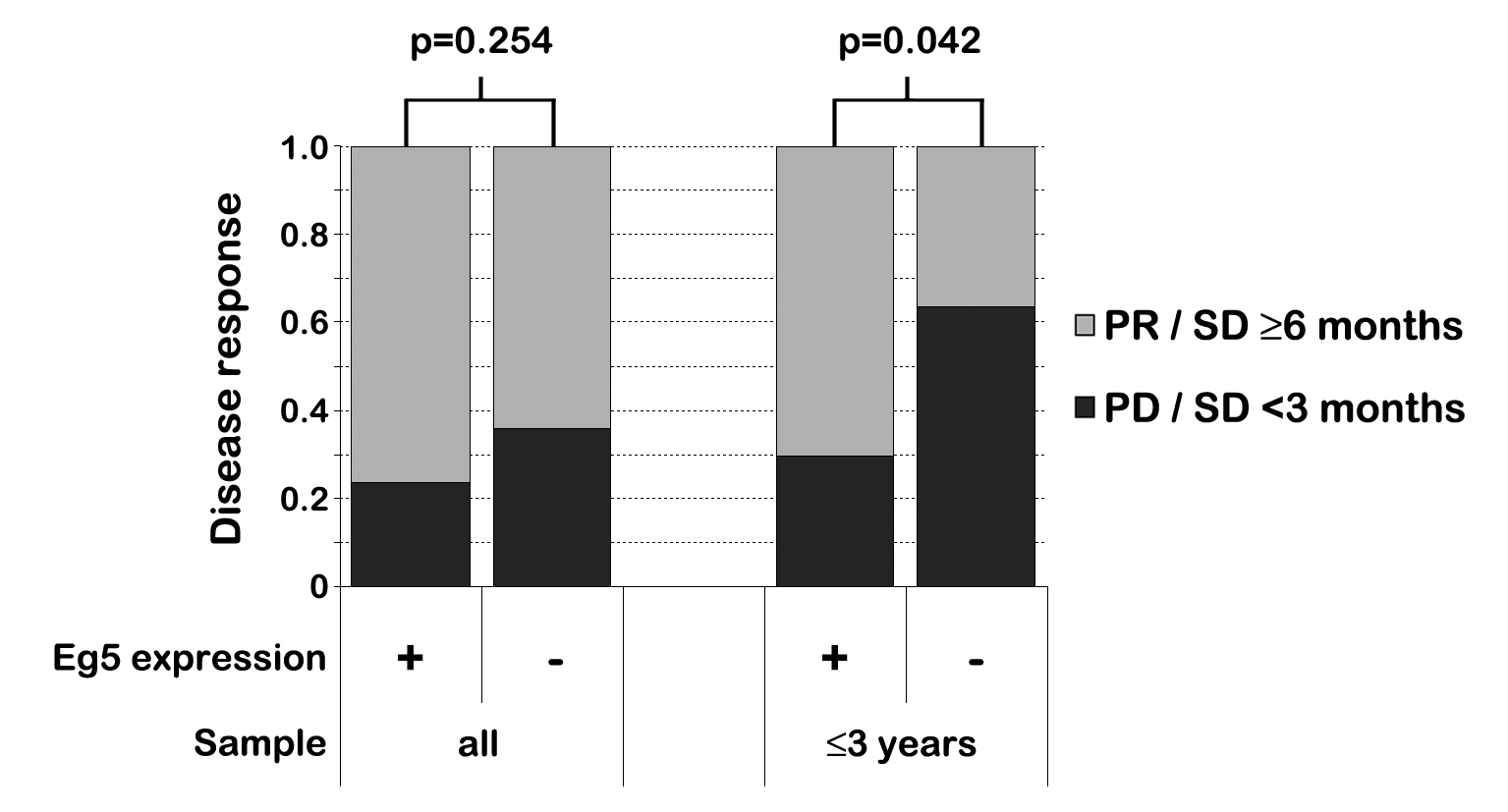
**

**Supplementary Figure 2.** Best disease response to docetaxel therapy in mCRPC patients, stratified by nuclear Eg5-expression of their tumor. Patients with a stable disease (SD) duration of three to six months were excluded from this analysis. The most recent PCa tissue before docetaxel therapy was analyzed from all patients (left) or only from patients who had tissue available obtained from the patient within three years before docetaxel therapy (right). In general, patients with nuclear Eg5-expression had a higher percentage of partial responses (PR) or extended SD (≥6 months). PD, progressive disease.

**
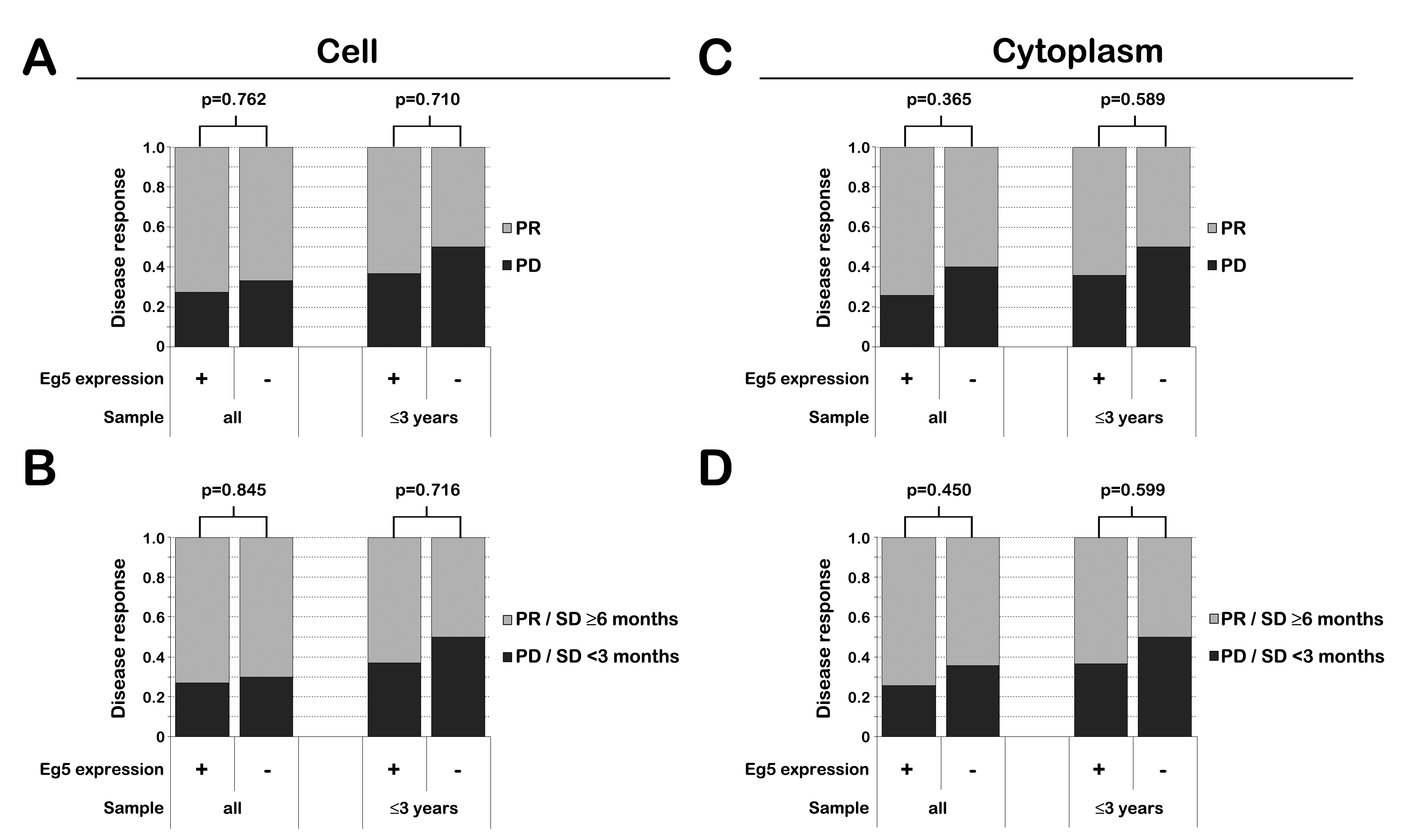
**

**Supplementary Figure 3.** Best disease response to docetaxel therapy in mCRPC patients, stratified by cellular (any compartment) (A-B) or cytoplasmic (C-D) Eg5-expression of their tumor. The most recent prostate cancer tissue before docetaxel therapy was analyzed from all patients (marked ‘all’ in each graph) or only from patients who had tissue available obtained from the patient within three years before docetaxel therapy (marked ‘≤3 years’ in each graph). No relationship was evident between cellular or cytoplasmic Eg5 expression and docetaxel response (p≥0.365). A. and C. Patients with stable disease (SD) were excluded from this analysis. B. and D. Patients who had a partial response (PR) or SD ≥6 months (prolonged SD), as well as patients who had progressive disease (PD) or SD <3 months were selected for this analysis.

**
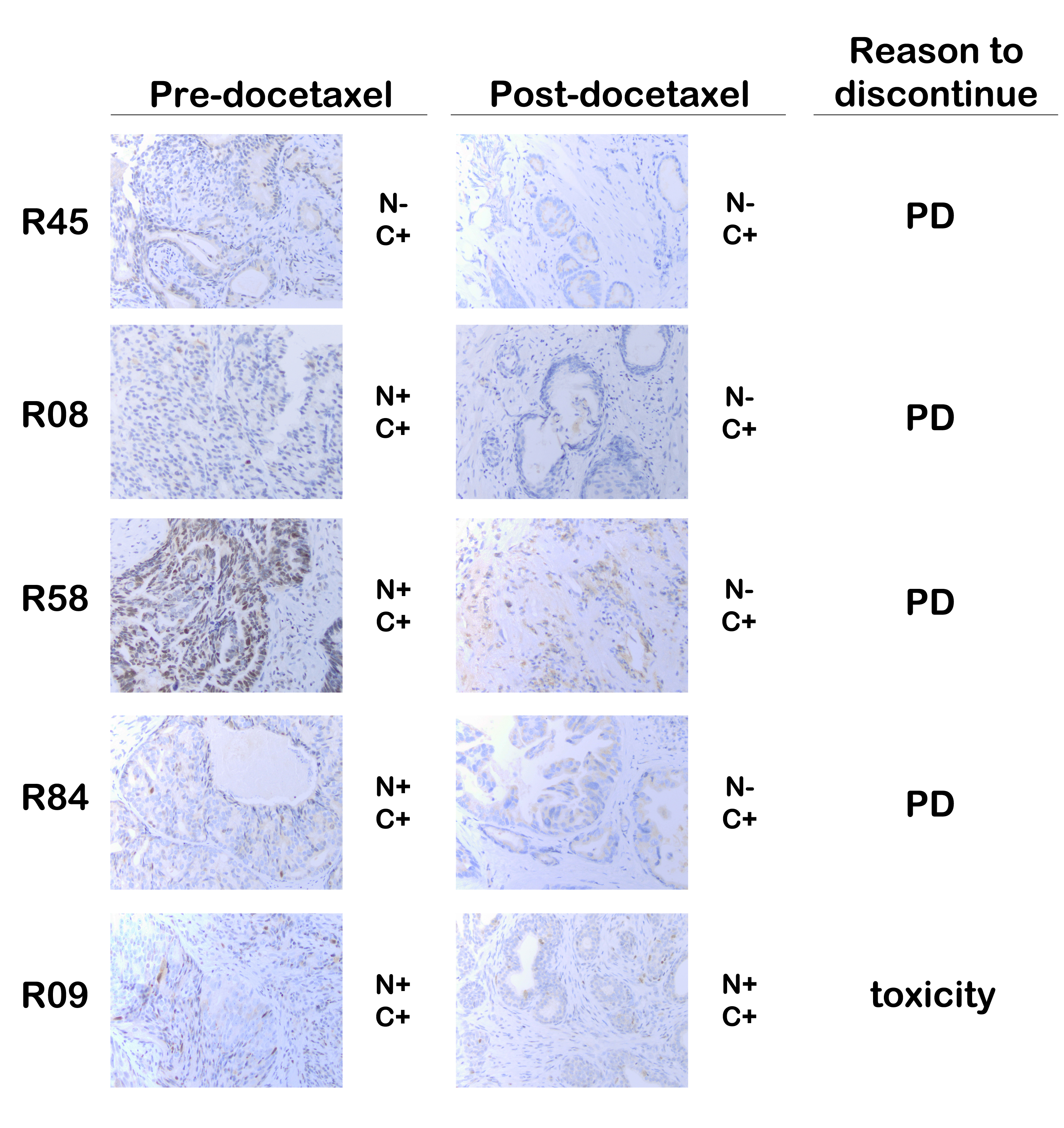
**

**Supplementary Figure 4.** Comparison of Eg5 expression in prostate cancer samples pre- and post-docetaxel. Cytoplasmic Eg5 expression did not alter in these patients, three out of four tumors with positive Eg5 nuclei before docetaxel therapy did not have nuclear Eg5 expression after docetaxel treatment. N+, positive nuclear staining; N-, negative nuclear staining; C+, positive cytoplasmic staining; C-, negative cytoplasmic staining; PD, progressive disease.

**Supplementary table 1: Correlation between Gleason-score, docetaxel-response and nuclear Eg5-expression**

|  |  | **Number of patients** | **Gleason [median (IQR)]** | | **p-value** |
| --- | --- | --- | --- | --- | --- |
| Patients with tissue available within 3 years before docetaxel | |  |  |  |  |
|  | Eg5-positive | 11 | 8 | (7-9) | 0.994 |
|  | Eg5-negative | 32 | 8 | (7-9) |
|  |  |  |  |  |  |
|  | Progressive disease | 16 | 8 | (8-9) | 0.884 |
|  | Partial response | 27 | 8 | (7-9) |
|  |  |  |  |  |  |
| All patients | |  |  |  |  |
|  | Progressive disease | 21 | 8 | (7-9) | 0.343 |
|  | Partial response | 49 | 8 | (7-9) |
